# Pembrolizumab versus paclitaxel for previously treated PD-L1-positive advanced gastric or gastroesophageal junction cancer: 2-year update of the randomized phase 3 KEYNOTE-061 trial

**DOI:** 10.1007/s10120-021-01227-z

**Published:** 2021-09-01

**Authors:** Charles S. Fuchs, Mustafa Özgüroğlu, Yung-Jue Bang, Maria Di Bartolomeo, Mario Mandala, Min-Hee Ryu, Lorenzo Fornaro, Tomasz Olesinski, Christian Caglevic, Hyun C. Chung, Kei Muro, Eric Van Cutsem, Anneli Elme, Peter Thuss-Patience, Ian Chau, Atsushi Ohtsu, Pooja Bhagia, Anran Wang, Chie-Schin Shih, Kohei Shitara

**Affiliations:** 1 Yale Cancer Center and Smilow Cancer Hospital, 333 Cedar Street, New Haven, CT 06510 USA; 2 Department of Internal Medicine, Division of Medical Oncology, Cerrahpaşa Medical Faculty, Istanbul University–Cerrahpaşa, Istanbul, Turkey; 3 Department of Internal Medicine, Seoul National University College of Medicine, Seoul, South Korea; 4 Department of Medical Oncology, Fondazione IRCCS Istituto Nazionale Tumori, Milan, Italy; 5 Unit of Medical Oncology, University of Perugia, Perugia, Italy; 6 Department of Oncology, Asan Medical Center, University of Ulsan College of Medicine, Seoul, South Korea; 7 Unit of Medical Oncology, Department of Translational Research and New Technology in Medicine and Surgery, Azienda Ospedaliero-Universitaria Pisana, Pisa, Italy; 8 Department of Oncological Gastroenterology, Maria Skłodowska–Curie Memorial, Warsaw, Poland; 9 Department of Cancer Research, Instituto Oncologico Fundacion Arturo Lopez, Santiago, Chile; 10 Division of Medical Oncology, Yonsei Cancer Center, Yonsei University College of Medicine, Seoul, South Korea; 11 Department of Clinical Oncology, Aichi Cancer Center Hospital, Nagoya, Japan; 12 Department of Digestive Oncology, University Hospitals Gasthuisberg Leuven and KU, Leuven, Belgium; 13 Chemotherapy Centre and Oncology and Hematology Clinic, The North Estonia Medical Centre, Tallinn, Estonia; 14 Medical Department, Division of Hematology, Oncology, and Tumor Immunology, Charité–University Medicine Berlin, Campus Virchow-Klinikum, Berlin, Germany; 15 Department of Medicine, Royal Marsden NHS Foundation Trust, London, UK; 16 Department of Gastroenterology and Gastrointestinal Oncology, National Cancer Center Hospital East, Kashiwa, Japan; 17 Department of Medical Oncology, Merck & Co., Inc, Kenilworth, NJ USA; 18 Department of Biostatistics and Research Decision Science, Merck & Co., Inc, Kenilworth, NJ USA

**Keywords:** Pembrolizumab, Chemotherapy, Gastric cancer, Gastroesophageal junction cancer

## Abstract

**Background:**

In the phase 3 KEYNOTE-061 study (cutoff: 10/26/2017), pembrolizumab did not significantly prolong OS vs paclitaxel as second-line (2L) therapy in PD-L1 combined positive score (CPS) ≥ 1 gastric/GEJ cancer. We present results in CPS ≥ 1, ≥ 5, and ≥ 10 populations after two additional years of follow-up (cutoff: 10/07/2019).

**Methods:**

Patients were randomly allocated 1:1 to pembrolizumab 200 mg Q3W for ≤ 35 cycles or standard-dose paclitaxel. Primary endpoints: OS and PFS (CPS ≥ 1 population). HRs were calculated using stratified Cox proportional hazards models.

**Results:**

366/395 patients (92.7%) with CPS ≥ 1 died. Pembrolizumab demonstrated a trend toward improved OS vs paclitaxel in the CPS ≥ 1 population (HR, 0.81); 24-month OS rates: 19.9% vs 8.5%. Pembrolizumab incrementally increased the OS benefit with PD-L1 enrichment (CPS ≥ 5: HR, 0.72, 24-month rate, 24.2% vs 8.8%; CPS ≥ 10: 0.69, 24-month rate, 32.1% vs 10.9%). There was no difference in median PFS among treatment groups (CPS ≥ 1: HR, 1.25; CPS ≥ 5: 0.98; CPS ≥ 10: 0.79). ORR (pembrolizumab vs paclitaxel) was 16.3% vs 13.6% (CPS ≥ 1), 20.0% vs 14.3% (CPS ≥ 5), and 24.5% vs 9.1% (CPS ≥ 10); median DOR was 19.1 months vs 5.2, 32.7 vs 4.8, and NR vs 6.9, respectively. Fewer treatment-related AEs (TRAEs) occurred with pembrolizumab than paclitaxel (53% vs 84%).

**Conclusion:**

In this long-term analysis, 2L pembrolizumab did not significantly improve OS but was associated with higher 24-month OS rates than paclitaxel. Pembrolizumab also increased OS benefit with PD-L1 enrichment among patients with PD-L1-positive gastric/GEJ cancer and led to fewer TRAEs than paclitaxel.

**Trial registration:**

ClinicalTrials.gov, NCT02370498

**Supplementary Information:**

The online version contains supplementary material available at 10.1007/s10120-021-01227-z.

## Introduction

Globally, gastric cancer is one of the most common and deadly cancers, with more than one million new cases diagnosed annually [1]. Many patients present with advanced-stage disease, for which second-line treatment options include single-agent chemotherapy with a taxane or irinotecan and the antivascular endothelial growth factor receptor 2 antibody ramucirumab, alone or combined with paclitaxel [2]. Pembrolizumab is a selective, humanized monoclonal antibody against programmed death 1 (PD-1) that prevents interaction between PD-1 and its ligands, PD-L1 and PD-L2 [3], and that has demonstrated antitumor activity and manageable safety in patients with advanced gastric or gastroesophageal (GEJ) cancer across multiple lines of therapy [4–8]. Based on data from cohort 1 of the KEYNOTE-059 study [9], pembrolizumab was approved in the United States for the treatment of patients with recurrent locally advanced or metastatic advanced gastric/GEJ adenocarcinoma expressing PD-L1 (combined positive score [CPS] ≥ 1) that progressed on at least two previous lines of therapy [3].

KEYNOTE-061 was a randomized, open-label, phase 3 trial of pembrolizumab compared with paclitaxel for previously treated advanced gastric/GEJ cancer [7]. In patients with CPS ≥ 1 tumors (data cutoff date: October 26, 2017), pembrolizumab did not significantly improve overall survival (OS) compared with paclitaxel (hazard ratio [HR], 0.82; 95% CI 0.66–1.03; one-sided *P* = 0.0421) or progression-free survival (PFS; HR, 1.27; 95% CI 1.03–1.57). Duration of response (DOR) was substantially longer with pembrolizumab than with paclitaxel (median, 18.0 vs 5.2 months), and pembrolizumab demonstrated a better safety profile than paclitaxel [7]. Herein we present results from KEYNOTE-061 based on two additional years of follow-up.

## Methods

### Study design

The study design for KEYNOTE-061 has been reported [7]. In brief, eligible patients had histologically or cytologically confirmed adenocarcinoma of the stomach or GEJ that was metastatic or locally advanced but unresectable, disease progression per Response Evaluation Criteria in Solid Tumors (RECIST) version 1.1 after first-line therapy with a platinum and fluoropyrimidine, and Eastern Cooperative Oncology Group performance status (ECOG PS) 0 or 1. Histology was evaluated by investigator. Patients were randomly allocated 1:1 to intravenous pembrolizumab 200 mg every 3 weeks for up to 2 years or paclitaxel 80 mg/m^2^ on days 1, 8, and 15 of each 4-week cycle or until disease progression, intolerable toxicity, physician decision, or patient withdrawal of consent. Randomization was stratified according to geographic region (Europe, Israel, North America, and Australia vs Asia vs rest of the world), time to progression on first-line therapy (< 6 months vs ≥ 6 months), and PD-L1 expression status (CPS < 1 vs ≥ 1). After 489 patients were enrolled, the independent data monitoring committee recommended that enrollment be restricted to patients with CPS ≥ 1 tumors on the basis of outcomes in patients with CPS < 1 tumors [7]. Consequently, all final 103 patients had CPS ≥ 1 tumors.

PD-L1 expression was assessed in archival or newly collected tumor samples at a central laboratory using PD-L1 IHC 22C3 pharmDx (Agilent) and measured using the CPS, defined as the number of PD-L1–staining cells (tumor cells, lymphocytes, macrophages) as a proportion of the total number of viable tumor cells, multiplied by 100.

The study protocol and all amendments were approved by the institutional review board or ethics committee at each institution. The study was conducted in accordance with the protocol and its amendments and Good Clinical Practice guidelines. All patients provided written informed consent before enrollment.

### Outcomes

The primary objectives of this analysis were OS (defined as the time from randomization to death from any cause) and PFS (defined as the time from randomization to radiologic disease progression assessed per RECIST v1.1 by masked and independent central review or death from any cause) in the population with CPS ≥ 1 tumors.

Additional exploratory objectives included OS and PFS in the populations with CPS ≥ 5 and CPS ≥ 10 tumors; response rate (defined as the proportion of patients with complete response [CR] or partial response [PR]) and DOR (defined as the time from first documented CR or PR to radiologic disease progression or death from any cause), both assessed per RECIST v1.1 by masked and independent central review and by investigator assessment in the populations with CPS ≥ 1, CPS ≥ 5, and CPS ≥ 10 tumors; and safety in all patients, irrespective of CPS.

### Statistical analysis

The analyses of the intention-to-treat population and the PD-L1 CPS ≥ 1 population were prespecified, whereas the analyses of the CPS ≥ 5 and CPS ≥ 10 subgroups were post hoc.

OS, PFS, and response rate were analyzed in the intention-to-treat population, defined as all patients who were randomly allocated to treatment, irrespective of whether they received the treatment. DOR was analyzed in all patients whose best response was CR or PR. Safety was assessed in all patients who received at least one dose of study treatment.

SAS version 9.4 (SAS Institute) was used for all statistical analyses. OS, PFS, and DOR were estimated using the Kaplan–Meier method. HRs and their associated 95% CIs were calculated using stratified Cox proportional hazards models with Efron’s method of tie handling. Kaplan–Meier analysis of OS was also analyzed in the protocol-specified subgroup of ECOG PS 0 or 1 based on the pembrolizumab treatment effect previously observed [7].

This trial is registered with ClinicalTrials.gov, NCT02370498.

## Results

**Fig. 1:**
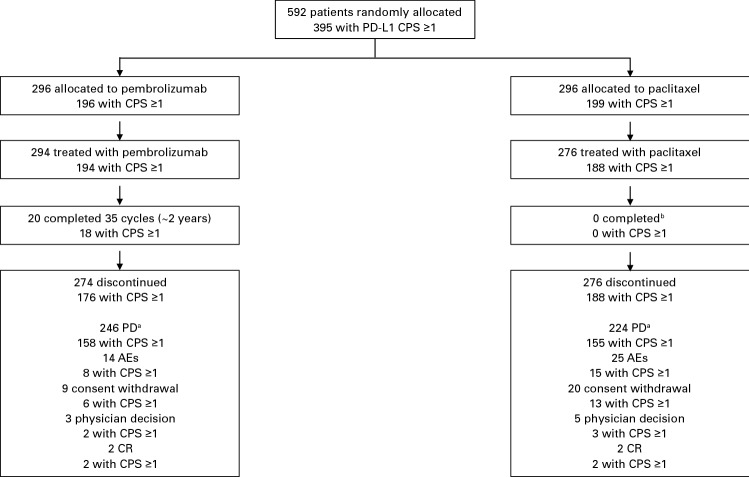
Patient disposition. *AE* adverse event, *CPS* combined positive score, *CR* complete response, *PD* progressive disease. ^a^Defined as clinical progression or progressive disease. ^b^There was no maximum number of doses of paclitaxel

The time from randomization to the data cutoff date of October 7, 2019, was 4 years and 4 months. At the time of analysis, 18 of 194 patients (9.3%) in the CPS ≥ 1 population completed 2 years of treatment with pembrolizumab, and the remaining 176 of 194 patients (90.7%) discontinued before the 2-year limit; all paclitaxel-treated patients had already discontinued treatment at the time of the protocol-specified analysis (Fig. 1). Baseline demographics and disease characteristics were generally balanced between treatment groups in the total population and in the CPS ≥ 1 population (Table 1). Baseline characteristics for the CPS < 1, CPS ≥ 5, and CPS ≥ 10 populations are reported in Online Resource 1; the prevalences of most characteristics for each population were comparable to those of the total population.

**Table 1:** Baseline characteristics in the overall and the PD-L1 CPS ≥ 1 intention-to-treat populations

	All patients		Patients with PD-L1 CPS ≥ 1	
	Pembrolizumab *n* = 296	Paclitaxel *n* = 296	Pembrolizumab *n* = 196	Paclitaxel *n* = 199
Age, median (range), years	62.5 (27–87)	60.0 (20–86)	64.0 (33–87)	61.0 (24–86)
Men, *n* (%)	202 (68.2)	208 (70.3)	146 (74.5)	140 (70.4)
Region, *n* (%)				
Europe, Israel, North America, and Australia	190 (64.2)	187 (63.2)	131 (66.8)	132 (66.3)
Asia	88 (29.7)	89 (30.1)	52 (26.5)	52 (26.1)
Rest of world	18 (6.1)	20 (6.8)	13 (6.6)	15 (7.5)
ECOG PS, *n* (%)				
0	127 (42.9)	137 (46.3)	88 (44.9)	92 (46.2)
1	169 (57.1)	158 (53.4)	108 (55.1)	106 (53.3)
2	0	1 (0.3)^a^	0	1 (0.5)^a^
Histology, *n* (%)				
Adenocarcinoma	235 (79.4)	233 (78.7)	159 (81.1)	158 (79.4)
Tubular adenocarcinoma	20 (6.8)	30 (10.1)	12 (6.1)	23 (11.6)
Signet-ring cell carcinoma, diffuse type	15 (5.1)	11 (3.7)	6 (3.1)	4 (2.0)
Other	25 (8.4)	22 (7.4)	18 (9.2)	14 (7.0)
Missing	1 (0.3)	0	1 (0.5)	0
Histologic subtype, *n* (%)				
Diffuse	86 (29.1)	65 (22.0)	52 (26.5)	40 (20.1)
Intestinal	44 (14.9)	74 (25.0)	30 (15.3)	49 (24.6)
Mixed	10 (3.4)	10 (3.4)	9 (4.6)	7 (3.5)
Unknown	155 (52.4)	147 (49.7)	104 (53.1)	103 (51.8)
Missing	1 (0.3)	0	1 (0.5)	0
Primary location, *n* (%)				
Stomach	207 (69.9)	200 (67.6)	134 (68.4)	126 (63.3)
GEJ	89 (30.1)	96 (32.4)	62 (31.6)	73 (36.7)
Previous gastrectomy, *n* (%)				
Total	45 (15.2)	51 (17.2)	30 (15.3)	32 (16.1)
Subtotal	31 (10.5)	42 (14.2)	19 (9.7)	26 (13.1)
Partial	30 (10.1)	19 (6.4)	18 (9.2)	13 (6.5)
None	190 (64.2)	184 (62.2)	129 (65.8)	128 (64.3)
PD-L1 CPS, *n* (%)				
≥ 1	196 (66.2)	199 (67.2)	196 (100)	199 (100)
< 1	99 (33.4)	96 (32.4)	0	0
Unknown	1 (0.3)	1 (0.3)	0	0
TTP on first-line therapy, *n* (%)				
< 6 months	186 (62.8)	182 (61.5)	126 (64.3)	129 (64.8)
≥ 6 months	110 (37.2)	114 (38.5)	70 (35.7)	70 (35.2)
HER2 positive, *n* (%)	48 (16.2)	62 (20.9)	36 (18.4)	41 (20.6)
Current disease stage, *n* (%)				
Metastatic	293 (99.0)	294 (99.3)	193 (98.5)	198 (99.5)
Locally advanced	3 (1.0)	2 (0.7)	3 (1.5)	1 (0.5)
Peritoneal metastasis, *n* (%)	82 (27.7)	84 (28.4)	50 (25.5)	49 (24.6)
Presence of ascites, *n* (%)	47 (15.9)	43 (14.5)	20 (10.2)	26 (13.1)
MSI status, *n* (%)				
MSI-H	15 (5.0)	12 (4.1)	13 (6.6)	11 (5.5)
Non-MSI-H	244 (82.4)	243 (82.1)	161 (82.1)	165 (82.9)
Unknown	37 (12.5)	41 (13.9)	22 (11.2)	23 (11.6)

*CPS* combined positive score, *ECOG PS* Eastern Cooperative Oncology Group performance status, *GEJ* gastroesophageal junction, *HER2* human epidermal growth factor receptor 2, *MSI-H* microsatellite stability–high, *TTP* time to progression

^a^ECOG PS was 0 during screening but increased to 2 at the time of random allocation; this patient did not receive study treatment

**Fig. 2:**
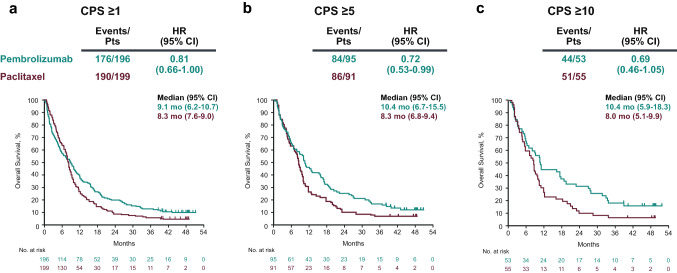
Kaplan–Meier analysis of overall survival in the populations with (**a**) CPS ≥ 1, (**b**) CPS ≥ 5, and (**c**) CPS ≥ 10 tumors. *CPS* combined positive score, *HR* hazard ratio, *Pts* patients

At the time of data cutoff, 366 patients in the CPS ≥ 1 population had died (176/196 [89.8%] in the pembrolizumab group and 190/199 [95.5%] in the paclitaxel group); median OS was 9.1 months (95% CI 6.2–10.7) for pembrolizumab and 8.3 months (95% CI 7.6–9.0) for paclitaxel (HR for death, 0.81; 95% CI 0.66–1.00) (Fig. 2a). The 24-month OS rates were 19.9% for pembrolizumab and 8.5% for paclitaxel. In the CPS ≥ 5 population, 170 of 186 patients (91.4%) had died (84/95 [88.4%] in the pembrolizumab group and 86/91 [94.5%] in the paclitaxel group); median OS was 10.4 months (95% CI 6.7–15.5) for pembrolizumab and 8.3 months (95% CI 6.8–9.4) for paclitaxel (HR for death, 0.72; 95% CI 0.53–0.99) (Fig. 2b). The 24-month OS rates were 24.2% for pembrolizumab and 8.8% for paclitaxel. In the CPS ≥ 10 population, 95 of 108 patients (88.0%) had died (44/53 [83.0%] in the pembrolizumab group and 51/55 [92.7%] in the paclitaxel group); median OS was 10.4 months (95% CI 5.9–18.3) for pembrolizumab and 8.0 months (95% CI 5.1–9.9) for paclitaxel (HR for death, 0.69; 95% CI 0.46–1.05) (Fig. 2c). The 24-month OS rates were 32.1% for pembrolizumab and 10.9% for paclitaxel.

**Fig. 3:**
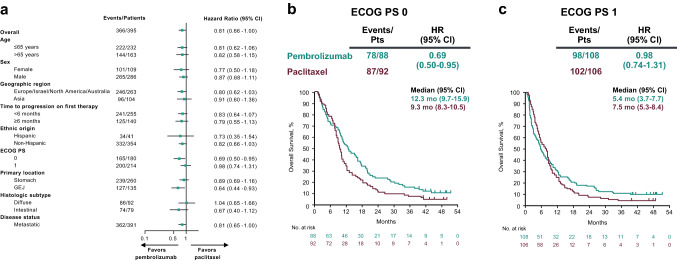
Overall survival analysis by (**a**) subgroups and Kaplan–Meier analysis in the population with CPS ≥ 1 tumors and ECOG PS (**b**) 0 or (**c**) 1. *CPS* combined positive score, *ECOG PS* Eastern Cooperative Oncology Group performance status, *GEJ* gastroesophageal junction, *HR* hazard ratio, *Pts* patients

In the CPS ≥ 1 population, OS favored pembrolizumab across all subgroups, with the exception of diffuse histology (Fig. 3a). We also performed a Kaplan–Meier analysis of OS based on ECOG PS. Among patients with CPS ≥ 1 tumors and ECOG PS 0, median OS was 12.3 months (95% CI 9.7–15.9) for pembrolizumab and 9.3 months (95% CI 8.3–10.5) for paclitaxel (HR for death, 0.69; 95% CI 0.50–0.95) (Fig. 3b); the 24-month OS rates were 23.9% and 10.9%, respectively. Among patients with CPS ≥ 1 tumors and ECOG PS 1, median OS was 5.4 months (95% CI 3.7–7.7) for pembrolizumab and 7.5 months (95% CI 5.3–8.4) for paclitaxel (HR for death, 0.98; 95% CI 0.74–1.31) (Fig. 3c); the 24-month OS rates were 16.7% and 6.6%, respectively. Factors affecting the treatment benefit in the overall population were explored with a similar subgroup analysis, shown in Online Resource 2.

**Fig. 4:**
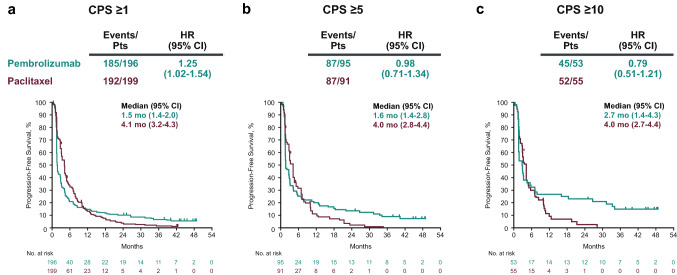
Kaplan–Meier analysis of progression-free survival in the populations with (**a**) CPS ≥ 1, (**b**) CPS ≥ 5, and (**c**) CPS ≥ 10 tumors. *CPS* combined positive score, *HR* hazard ratio, *Pts* patients

In the population with CPS ≥ 1 tumors, 377 of 395 patients (95.4%) experienced disease progression or died (185/196 [94.4%] in the pembrolizumab group and 192/199 [96.5%] in the paclitaxel group); median PFS was 1.5 months (95% CI 1.4–2.0) for pembrolizumab and 4.1 months (95% CI 3.2–4.3) for paclitaxel (HR for disease progression or death, 1.25; 95% CI 1.02–1.54) (Fig. 4a). In the population with CPS ≥ 5 tumors, 174 of 186 patients (93.5%) experienced disease progression or died (87/95 [91.6%] in the pembrolizumab group and 87/91 [95.6%] in the paclitaxel group); median PFS was 1.6 months (95% CI 1.4–2.8) for pembrolizumab and 4.0 months (95% CI 2.8–4.4) for paclitaxel (HR for disease progression or death, 0.98; 95% CI 0.71–1.34) (Fig. 4b). In the population with CPS ≥ 10 tumors, 97 of 108 patients (89.8%) experienced disease progression or died (45/53 [84.9%] in the pembrolizumab group and 52/55 [94.5%] in the paclitaxel group); median PFS was 2.7 months (95% CI 1.4–4.3) for pembrolizumab and 4.0 months (95% CI 2.7–4.4) for paclitaxel (HR for disease progression or death, 0.79; 95% CI 0.51–1.21) (Fig. 4c).

In the population with CPS ≥ 1 tumors, confirmed responses were observed in 32 of 196 patients in the pembrolizumab group (response rate, 16.3%) and in 27 of 199 patients in the paclitaxel group (response rate, 13.6%); CRs were observed in nine patients (4.6%) and five patients (2.5%), respectively (Table 2). In the population with CPS ≥ 5 tumors, confirmed responses were observed in 19 of 95 patients in the pembrolizumab group (response rate, 20.0%) and in 13 of 91 patients in the paclitaxel group (response rate, 14.3%); CRs were observed in seven patients (7.4%) and two patients (2.2%), respectively (Table 2). In the population with CPS ≥ 10 tumors, confirmed responses were observed in 13 of 53 patients in the pembrolizumab group (response rate, 24.5%) and in 5 of 55 patients in the paclitaxel group (response rate, 9.1%); CRs were observed in seven patients (13.2%) and one patient (1.8%), respectively (Table 2). Median DOR was longer in the pembrolizumab group than in the paclitaxel group, regardless of CPS status; median DOR in the pembrolizumab group increased with increasing PD-L1 enrichment (CPS ≥ 1, 19.1 months; CPS ≥ 5, 32.7 months; CPS ≥ 10, not reached) (Table 2). More than 60% of responders in the pembrolizumab group had responses lasting ≥ 12 months (Table 2).

**Table 2:** Response by PD-L1 combined positive score

	CPS ≥ 1		CPS ≥ 5		CPS ≥ 10	
	Pembrolizumab *n* = 196	Paclitaxel *n* = 199	Pembrolizumab *n* = 95	Paclitaxel *n* = 91	Pembrolizumab *n* = 53	Paclitaxel *n* = 55
ORR^a^, *n* (%)	32 (16.3)	27 (13.6)	19 (20.0)	13 (14.3)	13 (24.5)	5 (9.1)
CR	9 (4.6)	5 (2.5)	7 (7.4)	2 (2.2)	7 (13.2)	1 (1.8)
PR	23 (11.7)	22 (11.1)	12 (12.6)	11 (12.1)	6 (11.3)	4 (7.3)
SD, *n* (%)	44 (22.4)	90 (45.2)	23 (24.2)	42 (46.2)	12 (22.6)	28 (50.9)
PD, *n* (%)	95 (48.5)	46 (23.1)	45 (47.4)	20 (22.0)	23 (43.4)	11 (20.0)
Not available^b^	25 (12.8)	36 (18.1)	8 (8.4)	16 (17.6)	5 (9.4)	11 (20.0)
Median DOR^c^ (range), months	19.1 (1.4 + to 47.1 +)	5.2 (1.3 + to 16.8)	32.7 (4.1 to 47.1 +)	4.8 (1.3 + to 15.3)	NR (4.1 to 47.1 +)	6.9 (2.6 to 6.9)
Patients with extended response duration (≥ 12 months), *n* (%)	19 (61.3)	3 (29.5)	13 (68.4)	1 (12.3)	10 (76.9)	0

“ + ” indicates that there was no disease progression at the time of the last disease assessment

*CPS* combined positive score, *CR* complete response, *DOR* duration of response, *NR* not reached, *ORR* objective response rate, *PD* progressive disease, *PR* partial response, *RECIST* Response Evaluation Criteria in Solid Tumors, *SD* stable disease

^a^Response based on blinded independent central review per RECIST v1.1 with confirmation

^b^Not evaluable or no assessment

^c^From the Kaplan–Meier method for censored data

Treatment-related adverse events (AEs) occurred in 157 of 294 patients (53.4%) treated with pembrolizumab and 233 of 276 patients (84.4%) treated with paclitaxel (Table 3); grade 3–5 treatment-related AEs were reported in 44 of 294 patients (15.0%) and 97 of 276 patients (35.1%), respectively. The most common grade 3–5 treatment-related AEs (≥ 2% in either group) were fatigue and anemia in the pembrolizumab group and decreased neutrophil count, anemia, fatigue, neutropenia, decreased white blood cell count, and peripheral neuropathy in the paclitaxel group. Four patients died of treatment-related AEs (pembrolizumab,* n* = 3; paclitaxel,* n* = 1).

**Table 3:** Adverse events in the overall as-treated population

	Pembrolizumab *n* = 294		Paclitaxel *n* = 276	
	Any grade	Grade 3–5	Any grade	Grade 3–5
Related to treatment				
Any	157 (53.4)	44 (15.0)	233 (84.4)	97 (35.1)
Occurring in ≥ 10% in either group				
Fatigue	35 (11.9)	7 (2.4)	64 (23.2)	13 (4.7)
Decreased appetite	24 (8.2)	2 (0.7)	43 (15.6)	0
Nausea	17 (5.8)	1 (0.3)	50 (18.1)	2 (0.7)
Diarrhea	16 (5.4)	1 (0.3)	38 (13.8)	1 (0.4)
Anemia	10 (3.4)	7 (2.4)	41 (14.9)	13 (4.7)
Alopecia	1 (0.3)	0	111 (40.2)	3 (1.1)
Peripheral neuropathy	1 (0.3)	0	40 (14.5)	6 (2.2)
Neutrophil count decreased	0	0	35 (12.7)	28 (10.1)
Peripheral sensory neuropathy	0	0	35 (12.7)	3 (1.1)
Immune-mediated adverse events and infusion reactions				
Any	55 (18.7)	11 (3.7)	21 (7.6)	5 (1.8)
Hypothyroidism	24 (8.2)	0	1 (0.4)	0
Hyperthyroidism	12 (4.1)	0	1 (0.4)	0
Pneumonitis	8 (2.7)	2 (0.7)	0	0
Infusion reactions	5 (1.7)	0	13 (4.7)	1 (0.4)
Hepatitis	4 (1.4)	4 (1.4)	0	0
Hypophysitis	4 (1.4)	2 (0.7)	0	0
Colitis	3 (1.0)	1 (0.3)	4 (1.4)	3 (1.1)
Adrenal insufficiency	1 (0.3)	1 (0.3)	0	0
Severe skin reactions	1 (0.3)	1 (0.3)	1 (0.4)	0
Type 1 diabetes	1 (0.3)	0	0	0
Pancreatitis	0	0	1 (0.4)	1 (0.4)

Immune-mediated AEs and infusion reactions occurred in 55 of 294 patients (18.7%) treated with pembrolizumab and 21 of 276 patients (7.6%) treated with paclitaxel (Table 3). Grade 3–5 immune-mediated AEs occurring in two or more patients treated with pembrolizumab were hepatitis (*n* = 4), hypophysitis (*n* = 2), and pneumonitis (*n* = 2).

## Discussion

After approximately two additional years of follow-up in previously treated patients with gastric/GEJ cancer, the results of this long-term analysis from KEYNOTE-061 were consistent with those of the primary analysis [7]. However, second-line pembrolizumab did not significantly improve OS compared with paclitaxel at the primary analysis or after two additional years of follow-up. The Kaplan–Meier curve confirmed the beneficial treatment effects observed at 24 months in the primary analysis [7] after two additional years, with higher 24-month OS rates with pembrolizumab than with paclitaxel (19.9% vs 8.5%). Of note, the difference in 24-month OS rates between pembrolizumab and paclitaxel groups increased as the PD-L1 CPS cutoff increased (CPS ≥ 5, + 15.4%; CPS ≥ 10, + 21.3%), suggesting the utility of CPS to enrich for patients likely to benefit from pembrolizumab. Additionally, response rates were numerically higher with pembrolizumab, as indicated by two additional patients achieving CR (*n* = 9) compared with the primary analysis (*n* = 7). Durable responses were also observed after almost 4 years, some of which were ongoing at the data cutoff date. Of significance, the benefit of pembrolizumab in PFS and ORR also incrementally increased with PD-L1 enrichment. The safety profile for pembrolizumab remained consistent with that of the primary analysis and with that previously observed in other monotherapy trials, and no new safety signals were observed with long-term follow-up [7].

The observation that selection of patients by CPS enriched the long-term efficacy of pembrolizumab is consistent with previous clinical data in gastroesophageal cancer [10–12]. In the phase 3 CheckMate-649 study in patients with previously untreated, unresectable, non–HER2-positive gastric, GEJ, or esophageal adenocarcinoma, nivolumab plus chemotherapy resulted in significant improvements in OS and PFS vs chemotherapy alone in patients with PD-L1 CPS ≥ 5 (primary endpoint), patients with PD-L1 CPS ≥ 1, and all randomly assigned patients, and the treatment effect was more pronounced in the CPS ≥ 5 population. However, these studies, including KEYNOTE-061, were not powered to assess treatment effect specifically in the PD-L1 CPS < 1 population. In addition, during KEYNOTE-061, the independent data monitoring committee recommended that enrollment be restricted to patients with CPS ≥ 1 tumors, resulting in a small enrollment of patients with PD-L1 CPS < 1. With the available evidence, PD-L1 CPS remains valuable in deciding treatment strategies.

In the subgroup analysis of OS in patients with CPS ≥ 1 tumors, pembrolizumab-treated patients with ECOG PS 0 had a numerically longer 24-month OS rate than patients with ECOG PS 1 (23.9% vs 16.7%, respectively). These findings are also consistent with data reported at the primary analysis [7] and emphasize the need for further exploration in these patients.

Long-term data from immune checkpoint inhibitors in the second-line setting in patients with gastric/GEJ cancer are limited. In the phase 3 ATTRACTION-2 study, patients received third line or later nivolumab for advanced gastric/GEJ cancer [13]. After a median follow-up of 27 months, a higher 2-year OS rate was observed with nivolumab (10.6%) than with placebo (3.2%) in patients previously treated with at least two chemotherapy regimens; these findings were irrespective of PD-L1 status (assessed retrospectively on tumor cells using the 28–8 pharmDx assay). Although cross-trial comparisons should be interpreted with caution based on different patient populations and treatment lines, both the KEYNOTE-061 (more globally distributed in the second-line treatment setting) and the ATTRACTION-2 (predominantly Asian population [Japanese, South Korean, Taiwanese] in the third line or later treatment setting) studies suggested long-term OS benefits of anti–PD-1 therapy for patients with gastric/GEJ cancer.

As previously discussed [7], limitations of the study include its open-label design. As a result, there was an imbalance in the number of patients who were randomly allocated but who did not receive study treatment in the paclitaxel group compared with the pembrolizumab group. Consequently, patients in the paclitaxel group likely received other therapies, and this could have affected the study results and impacted the relative benefit of pembrolizumab vs paclitaxel. Subsequent therapy between the two treatment groups was also likely to be different, which could have affected the OS outcomes reported here. Furthermore, although the treatment groups were well balanced at baseline, the exclusion of patients whose tumors expressed CPS < 1 after 83% of patients were enrolled and the change in stratification factors after 21% of patients were enrolled might have introduced bias that affected the results [7].

Our findings suggest the potential for an increased treatment benefit with pembrolizumab monotherapy in patients with PD-L1 CPS ≥ 5 and CPS ≥ 10 tumors and in patients with better ECOG PS. Additionally, the safety profile of pembrolizumab remained favorable, showing fewer treatment-related AEs compared with paclitaxel. Taken together, these long-term data add insight to the existing body of evidence and support further exploration of pembrolizumab as monotherapy and as part of combination therapy in other gastric cancer settings.

## Supplementary Information

Below is the link to the electronic supplementary material.

Supplementary file1 (PDF 537 KB)

## Data Availability

Merck Sharp & Dohme Corp., a subsidiary of Merck & Co., Inc., Kenilworth, NJ, USA (MSD) is committed to providing qualified scientific researchers access to anonymized data and clinical study reports from the company’s clinical trials for the purpose of conducting legitimate scientific research. MSD is also obligated to protect the rights and privacy of trial participants and, as such, has a procedure in place for evaluating and fulfilling requests for sharing company clinical trial data with qualified external scientific researchers. The MSD data-sharing website (available at: http://engagezone.msd.com/ds_documentation.php) outlines the process and requirements for submitting a data request. Applications will be promptly assessed for completeness and policy compliance. Feasible requests will be reviewed by a committee of MSD subject matter experts to assess the scientific validity of the request and the qualifications of the requestors. In line with data privacy legislation, submitters of approved requests must enter into a standard data-sharing agreement with MSD before data access is granted. Data will be made available for request after product approval in the US and EU or after product development is discontinued. There are circumstances that may prevent MSD from sharing requested data, including country or region-specific regulations. If the request is declined, it will be communicated to the investigator. Access to genetic or exploratory biomarker data requires a detailed, hypothesis-driven statistical analysis plan that is collaboratively developed by the requestor and MSD subject matter experts; after approval of the statistical analysis plan and execution of a data-sharing agreement, MSD will either perform the proposed analyses and share the results with the requestor or will construct biomarker covariates and add them to a file with clinical data that is uploaded to an analysis portal so that the requestor can perform the proposed analyses.
