# Supplementary material for: Pembrolizumab versus paclitaxel for previously treated PD-L1-positive advanced gastric or gastroesophageal junction cancer: 2-year update of the randomized phase 3 KEYNOTE-061 trial
Source: Gastric Cancer. 2021 Sep 1;25(1):197–206. doi: 10.1007/s10120-021-01227-z (PMC8732941; doi:10.1007/s10120-021-01227-z)

Gastric Cancer

Pembrolizumab versus paclitaxel for previously treated PD-L1–positive advanced gastric or gastroesophageal junction cancer: 2-year update of the randomized phase 3 KEYNOTE-061 trial

Charles S. Fuchs,* Mustafa Özgüroğlu, Yung-Jue Bang, Maria Di Bartolomeo, Mario Mandala, Min-Hee Ryu, Lorenzo Fornaro, Tomasz Olesinski, Christian Caglevic, Hyun C. Chung, Kei Muro, Eric Van Cutsem, Anneli Elme, Peter Thuss-Patience, Ian Chau, Atsushi Ohtsu, Pooja Bhagia, Anran Wang, Chie-Schin Shih, Kohei Shitara

*Corresponding author

Yale Cancer Center, Smilow Cancer Hospital

Email: Charles.S.Fuchs@gmail.com

Online Resource 1. Baseline characteristics in the PD-L1 CPS <1, CPS ≥5, and CPS ≥10 populations

	Patients with PD-L1 CPS <1		Patients with PD-L1 CPS ≥5		Patients with PD-L1 CPS ≥10	
	**Pembrolizumab** ***n*** **= 99**	**Paclitaxel** ***n*** **= 96**	**Pembrolizumab** ***n*** **= 95**	**Paclitaxel** ***n*** **= 91**	**Pembrolizumab** ***n*** **= 53**	**Paclitaxel** ***n*** **= 55**
Age, median (range), years	59.0 (27-79)	59.0 (20-83)	64.0 (35-80)	59.0 (24-81)	66.0 (35-79)	60 (37-76)
Men, *n* (%)	55 (55.6)	68 (70.8)	66 (69.5)	61 (67.0)	35 (66.0)	35 (63.6)
Region, *n* (%)						
Europe, Israel, North America, and Australia	59 (59.6)	55 (57.3)	64 (67.4)	61 (67.0)	32 (60.4)	35 (63.6)
Asia	35 (35.4)	36 (37.5)	25 (26.3)	21 (23.1)	16 (30.2)	13 (23.6)
Rest of world	5 (5.1)	5 (5.2)	6 (6.3)	9 (9.9)	5 (9.4)	7 (12.7)
ECOG PS, *n* (%)						
0	39 (39.4)	45 (46.9)	44 (46.3)	40 (44.0)	24 (45.3)	24 (43.6)
1	60 (60.6)	51 (53.1)	51 (53.7)	51 (56.0)	29 (54.7)	31 (56.4)
Histology, *n* (%)						
Adenocarcinoma	76 (76.8)	74 (77.1)	78 (82.1)	70 (76.9)	44 (83.0)	43 (78.2)
Tubular adenocarcinoma	8 (8.1)	7 (7.3)	4 (4.2)	13 (14.3)	2 (3.8)	8 (14.5)

Signet-ring cell carcinoma, diffuse type	9 (9.1)	7 (7.3)	1 (1.1)	1 (1.1)	0	1 (1.8)
Other	6 (6.1)	8 (8.3)	12 (12.6)	7 (7.7)	7 (13.2)	3 (5.5)
Histologic subtype, *n* (%)						
Diffuse	34 (34.3)	24 (25.0)	31 (32.6)	20 (22.0)	17 (32.1)	10 (18.2)
Intestinal	14 (14.1)	25 (26.0)	14 (14.7)	19 (20.9)	6 (11.3)	11 (20.0)
Mixed	1 (1.0)	3 (3.1)	6 (6.3)	1 (1.1)	4 (7.5)	1 (1.8)
Unknown	0	0	44 (46.3)	51 (56.0)	26 (49.1)	33 (60.0)
Primary location, *n* (%)						
Stomach	72 (72.7)	73 (76.0)	69 (72.6)	60 (65.9)	35 (66.0)	35 (63.6)
GEJ	27 (27.3)	23 (24.0)	26 (27.4)	31 (34.1)	18 (34.0)	20 (36.4)
Previous gastrectomy, *n* (%)						
Total	15 (15.2)	19 (19.8)	20 (21.1)	16 (17.6)	14 (26.4)	7 (12.7)
Subtotal	12 (12.1)	15 (15.6)	13 (13.7)	15 (16.5)	8 (15.1)	12 (21.8)
Partial	12 (12.1)	6 (6.3)	9 (9.5)	5 (5.5)	4 (7.5)	4 (7.3)
None	60 (60.6)	56 (58.3)	53 (55.8)	55 (60.4)	27 (50.9)	32 (58.2)
TTP on first-line therapy, *n* (%)						
<6 months	60 (60.6)	53 (55.2)	62 (65.3)	57 (62.6)	36 (67.9)	36 (65.5)

≥6 months	39 (39.4)	43 (44.8)	33 (34.7)	34 (37.4)	17 (32.1)	19 (34.5)
HER2 positive, *n* (%)	12 (12.1)	21 (21.9)	19 (20.0)	15 (16.5)	10 (18.9)	9 (16.4)
Current disease stage, *n* (%)						
Metastatic	99 (100)	95 (99.0)	95 (100)	90 (98.9)	53 (100)	55 (100)
Locally advanced	0	1 (1.0)	0	1 (1.1)	0	0
Peritoneal metastasis, *n* (%)	31 (31.3)	35 (36.5)	26 (27.4)	23 (25.3)	11 (20.8)	14 (25.5)
Presence of ascites, *n* (%)	27 (27.3)	17 (17.7)	10 (10.5)	8 (8.8)	6 (11.3)	7 (12.7)
MSI status, *n* (%)						
MSI-H	2 (2.0)	1 (1.0)	9 (9.5)	6 (6.6)	8 (15.1)	5 (9.1)
Non-MSI-H	82 (82.8)	78 (81.2)	79 (83.2)	76 (83.5)	41 (77.4)	44 (80.0)
Unknown	15 (15.2)	17 (17.7)	7 (7.4)	9 (9.9)	4 (7.5)	6 (10.9)

CPS, combined positive score; ECOG PS, Eastern Cooperative Oncology Group performance status; GEJ, gastroesophageal junction; HER2, human epidermal growth factor receptor 2; TTP, time to progression.

Online Resource 2. Overall survival analysis by subgroups in the overall population

	Events/Patients	Hazard Ratio (95% CI)
**Overall**	554/592	0.92 (0.78-1.10)
**Age**		
≤65 years	357/371	0.95 (0.77-1.19)
>65 years	197/221	0.93 (0.69-1.25)
**Sex**		
Female	170/182	0.98 (0.70-1.36)
Male	384/410	0.94 (0.76-1.16)
**Geographic region**		
Europe/Israel/North America/Australia	357/377	0.89 (0.72-1.11)
Asia	163/177	1.04 (0.76-1.42)
Rest of world	34/38	0.69 (0.33-1.45)
**Time to progression on first therapy**		
<6 months	351/368	0.93 (0.75-1.16)
≥6 months	203/224	0.91 (0.68-1.20)
**PD-L1**		
Positive	366/395	0.81 (0.66-1.00)
Negative	188/197	1.18 (0.88-1.59)
**Ethnic origin**		
Hispanic	44/51	0.64 (0.32-1.29)
Non-Hispanic	510/541	0.94 (0.78-1.12)
**ECOG PS**		
0	241/264	0.77 (0.59-1.00)
1	312/327	1.14 (0.90-1.44)
**Primary location**		
Stomach	378/407	1.02 (0.83-1.26)
GEJ	176/185	0.72 (0.52-0.99)
**Histologic subtype**		
Diffuse	145/151	1.04 (0.72-1.51)
Intestinal	108/118	0.86 (0.56-1.32)
**Disease status**		
Metastatic	549/587	0.92 (0.78-1.10)

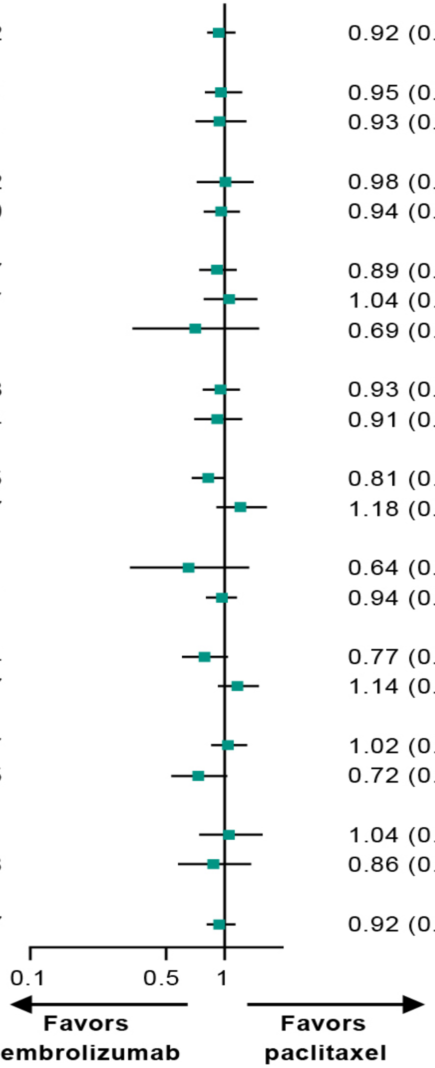

Supplement: Supplementary file 1 — Supplementary file1 (PDF 537 KB) [file 10120_2021_1227_MOESM1_ESM.pdf]
